# The First Report of Fetal Alcohol Effect in a 12 Year-Old Child in Korea

**DOI:** 10.4306/pi.2009.6.1.50

**Published:** 2009-03-31

**Authors:** Soo Young Bhang, Dong Hyun Ahn, Young Jin Lee, Ho Young An, Joon Ho Ahn

**Affiliations:** 1 Department of Psychiatry, University of Ulsan College of Medicine, Ulsan University Hospital, Ulsan, Korea.; 2 Department of Psychiatry, College of Medicine, Hanyang University, Seoul, Korea.; 3 Department of Psychiatry, University of Ulsan College of Medicine, Asan Medical Center, Seoul, Korea.

**Keywords:** Fetal alcohol spectrum disorder, Fetal alcohol effect, Attention deficit/hyperactivity disorder, Pregnancy, Seizure, Growth retardation

## Abstract

We present the first report of fetal alcohol effect in a 12 year-old child in Korea. The mother had consumed 162 g of alcohol per week continuously during pregnancy. His first febrile seizure occurred before he was 1 year old, and became more frequent 2 years later. He started showing signs of right paraplegia when he was 3.5 years old and brain MRI revealed periventricular leucomalacia near the left ventricle. He was microcephalic and his growth was retarded. He was irritable, impatient, impulsive, and inattentive, and showed disinterest in school activities and aggressive and dangerous behavior. After the diagnosis of attention deficit/hyperactivity disorder was made, psychopharmacological treatment and family support was initiated. After 10 months, he still had intermittent ideas of reference, although the aggressive behavior, inattentiveness, and impulsivity had improved. Using this case study, we stress the importance of maternal alcohol history in patients with these characteristics.

## Introduction

Fetal alcohol exposure affects the growth and development of neurons. Lemoine et al. first reported on the relationship between alcohol consumption during pregnancy and fetal development in 1968,^1^ but it was not until the reports of Jones et al. and Jones and Smith^2^,^3^ in 1973 that fetal alcohol spectrum disorder (FASD) became widely known. The prevalence of FASD in the United States is 0.5-2 per 1,000 births.^4^

In 2005, after analyzing the 1996 records of the Institute of Medicine Criteria, Hoyme et al.^5^ reported the feasibility of classifying FASD into five categories and suggested that cases without facial anomalies be referred to as alcohol-related neurodevelopmental disorder (ARND). The diagnostic criteria are given in Table 1.

In Korea, two cases of FASD have been reported,^6^,^7^ but none of Fetal Alcohol Effect (FAE; i.e., FASD without facial anomalies). We present the case of a 6^th^ grade patient with FAE exhibiting poliomyelitis, epilepsy, microcephaly, growth deficit, impulsivity, and pica.

## Case

**FIGURE 1:**
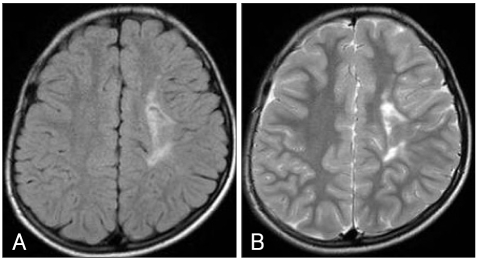
FLAIR and T2-weighted brain MR image. A and B show a high signal intensity lesion on FLAIR and T2-weighted brain MR image, highly suggestive of periventricular leukomalacia, in the left corona radiata and centrum semiovale (Taken when the patient was 10 years old). FLAIR: fluid attenuated inversion recovery.

We report a 12-year-old boy with FAE who was initially diagnosed with attention deficit/hyperactivity disorder (ADHD), but had an atypical course with severe aggression and acting out. His mother was in her late 30s and had consumed 162 g of alcohol (six bottles of rice wine) a week continuously during pregnancy. The patient had a normal, full-term, vaginal delivery and weighed 2.5 kg at birth. He ate cotton from his bedclothes frequently. The patient had symptoms of tiptoe gait at the age of 3 and br ain magnetic resonance imaging (MRI) revealed periventricular leukomalacia near the left ventricle compatible with cerebral palsy. The MRI findings persisted at several follow-up examinations (Figure 1). His first febrile seizure occurred before he was 1 year old. They became increasingly frequent along with general and partial seizures after the age of 3 years, sometimes occurring over seven times a day when he was 6 years old. Electroencephalography (EEG) showed partial seizures due to epileptiform discharge in the right frontocentral area. He was left-handed, microcephalic (2 SD below the mean) and his height and body weight were below the third percentile.

He was irritable, scratched tables with knives and pens when nervous, and threw desks and chairs when provoked during his preschool period. He showed no interest in activities in nursery school or kindergarten. He started shop-lifting during the third grade and ran away from home three times. He was inattentive in class, had no interest in studying, and his aggressive behavior hindered his classmates. The patient was referred to the psychiatry department by a child neurologist for behavioral problems. His first visit was on July 18, 2007. He scored 30 (>98^th^ percentile) on the Korean ADHD Rating Scale (K-ARS) for parents^8^ and 94 on the K-scale for the diagnosis of Internet addiction in Korea,^9^ which placed him in the high-risk group for Internet addiction and social dysfunction.

**FIGURE 2:**
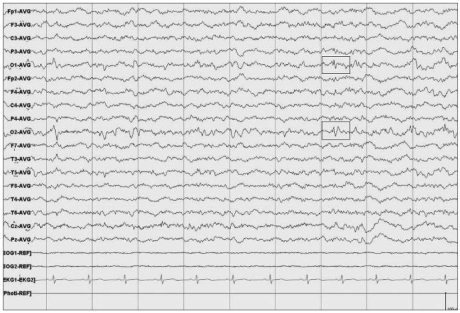
Electroencephalogram (ECG). The main feature of this recording is intermittent low amplitude spike discharges on both occipital areas (O1, O2). This record is suggestive of idiopathic partial epilepsy.

**FIGURE 3:**
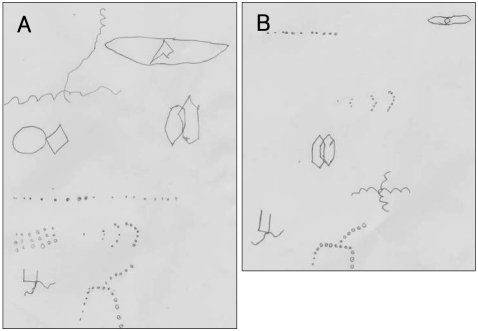
Bender-Gestalt Test (BGT). A: Copy. B: Recall. There is Curvature difficulty, Change in Angulation, distortion, slope and small size error. These findings suggest the developmental delay of visuomotor coordination and visuospatial function, the organic dysfunction of the brain, impaired planning, impulsivity and tendency of acting out.

Treatment was started with 10 mg of fluoxetine, 1 mg of risperidone, and 10 mg of methylphenidate CD. During outpatient treatment, although his emotional control improved, he was still involved in fights frequently, and in December 2007, tried to strangle his grandmother and stabbed a classmate with a small knife. He was hospitalized for 32 days. During that period, EEG showed intermittent low-amplitude discharges in both occipital regions (O1, O2), which is characteristic of an interictal period (Figure 2). Neuropsychological testing indicated that his full-scale IQ measured using the Korean Educational Development Institute-Wechsler Intelligence Scale for children (KEDI-WISC IQ)^10^ was 84 (verbal 87, nonverbal 84) and he showed large variation between nonverbal tests. In addition, curvature difficulty, change in angulation, distortion, slope, and small size error on the Bender-Gestalt test (BGT) were observed (Figure 3). These findings suggest organic dysfunction of the brain, developmental delay in visuomotor coordination and visuospatial function, impaired planning, impulsivity, and tendency to act out. The continuous performance test^11^ and a commercial version called the Attention Deficit/Hyperactivity Disorder Diagnostic System (ADS)^12^ showed that the T scores of almost all parameters, such as omission errors, commission errors, reaction time, and standard deviation of the reaction time, were above 70, which means attention deficit and impulsivity were severe. The completion time of the Children's Color Trails Test (CCTT)^13^ was at the 12^th^ percentile and that of CCTT-2 and the score of interference B were below the 1^st^ percentile. The results of the Matching Familiar Figures Test for Korean Children (MFFTKC) showed that the "reaction time" was in the 30-70^th^ percentile and "error" was above the 70^th^ percentile, which means that the child was inattentive and impulsive.

He received both psychopharmacotherapy and behavioral modification therapy. One month after admission, he was discharged on 1 mg of risperidone, 800 mg of valproate and 20 mg of methylphenidate CD. He started to participate in classroom activities and he comes home early and no longer wanders around outside.

## Discussion

Burd et al.^14^ reported that FASD has a high rate of comorbid disorders, with 40% having ADHD, 15-20% mental retardation, 25% learning disorders, 4% cerebral palsy, and 8-10% epilepsy. In a study carried out in Norway,^15^,^16^ all of the patients with FASD had comorbid ADHD. They had problems with emotional control and schoolwork, and exhibited insufficient social interaction. Our patient had all of these problems and due to poor academic performance, aggressive behavior, insufficient sociality, and difficulties in school. His intelligence was low average, and he showed large variation in performance between nonverbal tests. Inattentiveness and impulsivity were suggested, and his visuomotor coordination and visuospatial functions were low for his age.

This patient had EEG findings of partial seizures for many years. In 1968, Paul Lemoine^1^ reported that in cases of FASD with abnormal EEGs, the abnormalities indicate immaturity of the brain. Synchronicity of EEG waves during the neonatal and infant period was reported in 1977,^17^ and the relationship between the heritability of the spectral power of the EEG and the effects of alcohol^18^ have also been reported. Recent studies report that 8-10% of patients with FASD have epilepsy.^14^ In addition, in 1998, O'Malley and Barr reported^19^ that of patients with FASD, 21% have temporal lobe epilepsy of the nondominant hemisphere, or both, and showed distinct Q waves. The EEG findings of temporal lobe epilepsy (TLE) and partial seizures in our patient agree with these findings.

In this case, the periventricular leucomalacia near the left ventricle was the most likely cause of the poliomyelitis.^20^ In this case, the effects of alcohol might have caused the symptoms of cerebral palsy, as the patient was not premature at birth. Generally, the cutoff value for growth retardation is the third percentile in height, weight, and head circumference. All three measurements were below the first percentile, and he was as small as a kindergarten student, which agrees with prior reports.^14^

In patients with FASD, early detection and diagnosis facilitate early intervention, and help find symptoms and probable causes for the deficit.^14^ Treatment requires a multidisciplinary team effort,^14^ and in our patient's case, the active cooperation of schoolteachers, home visits by a social worker, and assistance from hospital staff are all helping the grandmother care for the patient.

In Korea, the prevalence of female drinking jumped from 15.4% in 1995 to 32.1% in 2001.^21^ Adolescent drinking is also increasing rapidly.^22^ Therefore, the prevalence of FASD is also likely to be rapidly increasing, although warnings and advice for the general public are severely insufficient. We hope that this case helps other clinicians identify, diagnose, and treat other patients with FASD.

## Figures and Tables

**TABLE 1:**
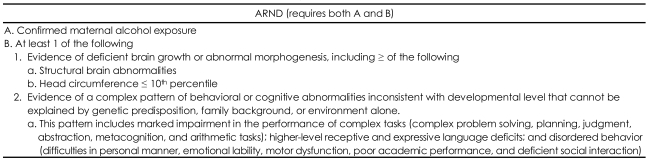
Proposed Clarification of the 1996 IOM criteria for Diagnosis of ARND^5^

IOM: Institute of Medicine, ARND: alcohol-related neurodevelopmental disorder
